# Supplementary material for: Remarkable recent changes in the genetic diversity of the avirulence gene AvrStb6 in global populations of the wheat pathogen Zymoseptoria tritici
Source: Mol Plant Pathol. 2021 Jul 14;22(9):1121–33. doi: 10.1111/mpp.13101 (PMC8358995; doi:10.1111/mpp.13101)
Supplement: Supplementary file 1 — FIGURE S1 Frequency of each of the identified AvrStb6 haplotypes along with their geographic origin [file MPP-22-1121-s008.pdf]

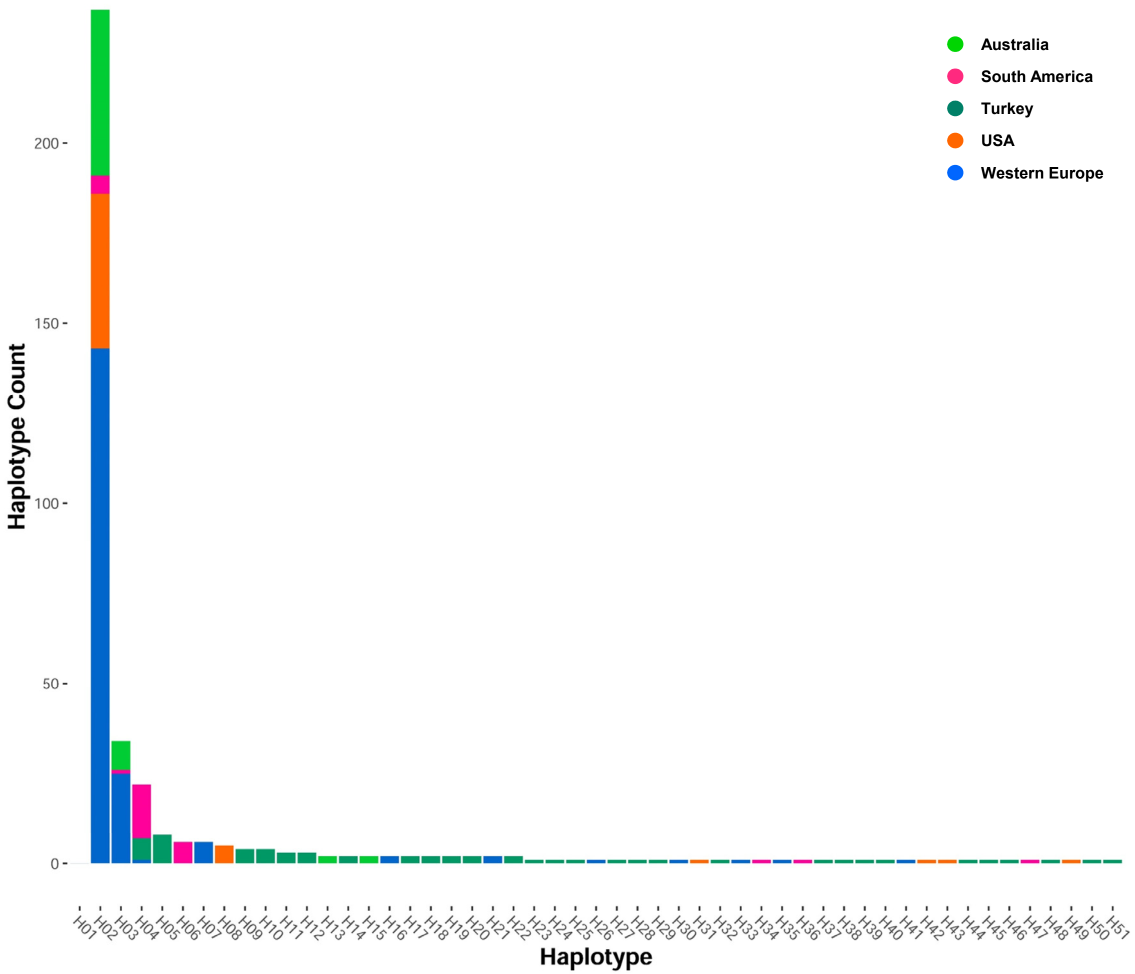

Figure S1. Frequency of each of the identified *AvrStb6* haplotypes along with their geographic origin.
