# Supplementary material for: Remarkable recent changes in the genetic diversity of the avirulence gene AvrStb6 in global populations of the wheat pathogen Zymoseptoria tritici
Source: Mol Plant Pathol. 2021 Jul 14;22(9):1121–33. doi: 10.1111/mpp.13101 (PMC8358995; doi:10.1111/mpp.13101)
Supplement: Supplementary file 2 — FIGURE S2 Alignment of AvrStb6 isoforms from Zymoseptoria tritici isolates tested in pathoassays to determine virulence on Stb6‐containing wheat [file MPP-22-1121-s003.pdf]

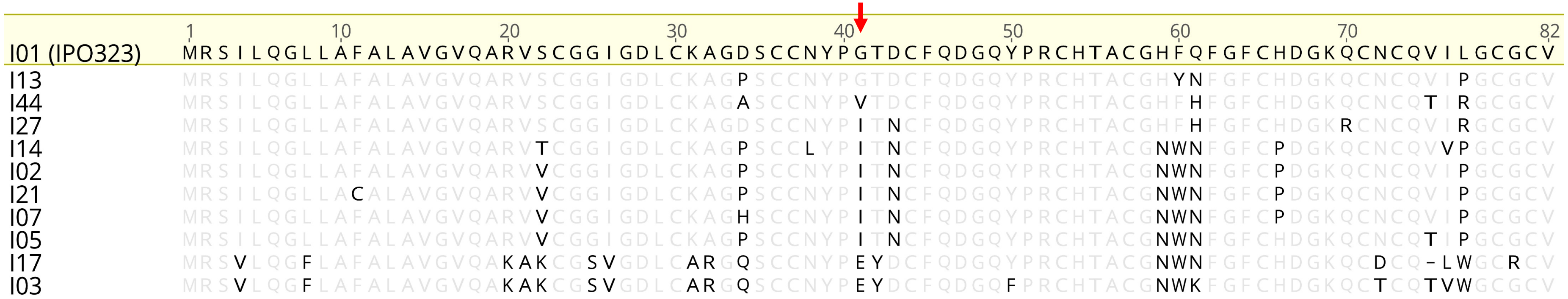

Figure S2. Alignment of AvrStb6 isoforms from *Z. tritici* isolates tested in pathoassays to determine virulence on *Stb6* containing wheat. Sequences were aligned using MAFFT v7.388. Of the above isoforms, all isolates were found to be virulent on *Stb6* containing wheat, with the exception of isolates possessing I01 (from reference isolate IPO323) and I13. Amino acids synonymous to the I01 reference sequence from isolate IPO323 are greyed. Missing residues relative to the reference are represented as dashes. The red arrow indicates the amino acid position #41, variation at which is hypothesised to determine the virulence/avirulence phenotype.
