# Supplementary figures and images for: Remarkable recent changes in the genetic diversity of the avirulence gene AvrStb6 in global populations of the wheat pathogen Zymoseptoria tritici
Source: Mol Plant Pathol. 2021 Jul 14;22(9):1121–33. doi: 10.1111/mpp.13101 (PMC8358995; doi:10.1111/mpp.13101)

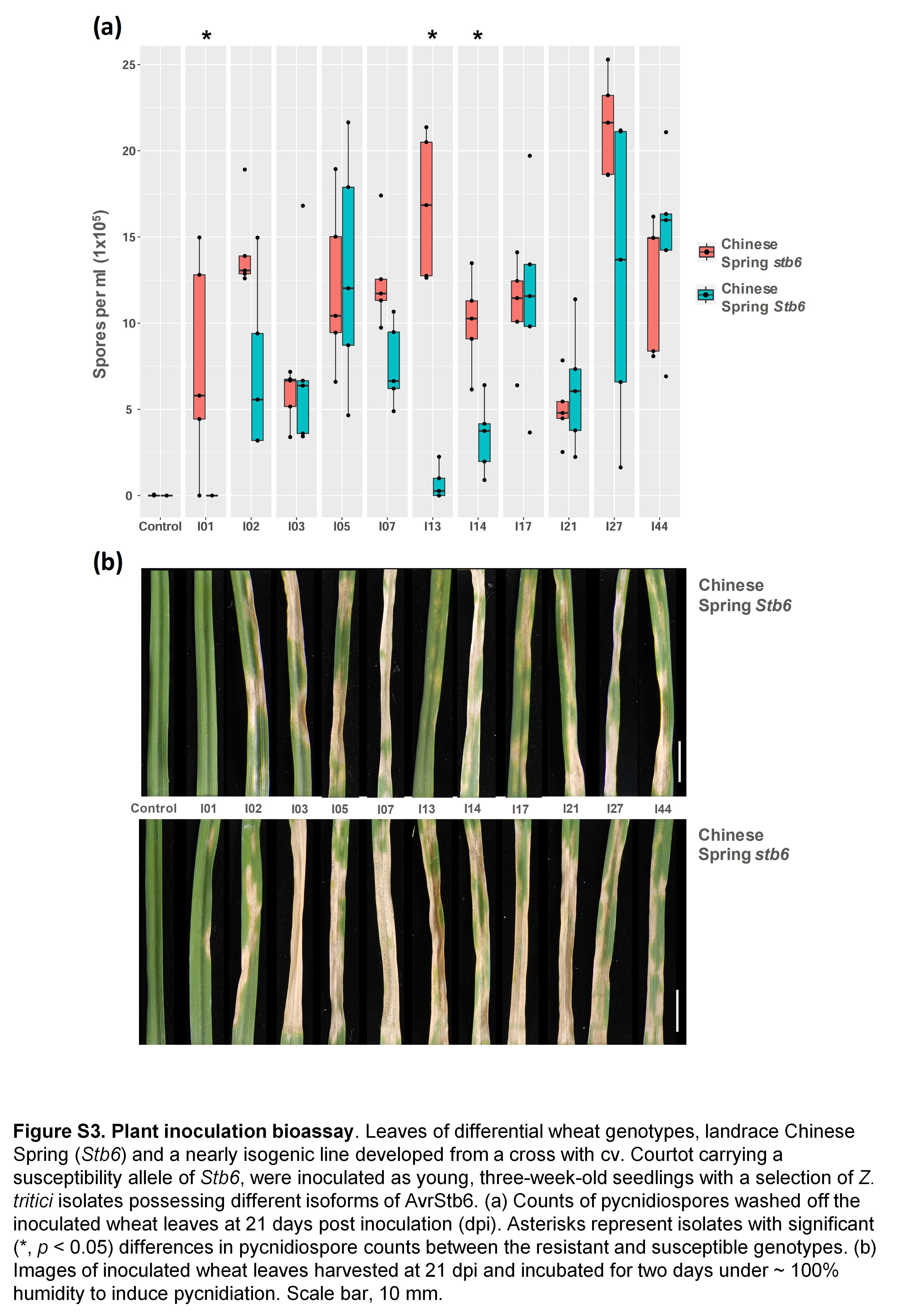

Supplement: Supplementary file 3 — FIGURE S3 Plant inoculation bioassay [file MPP-22-1121-s004.jpg]

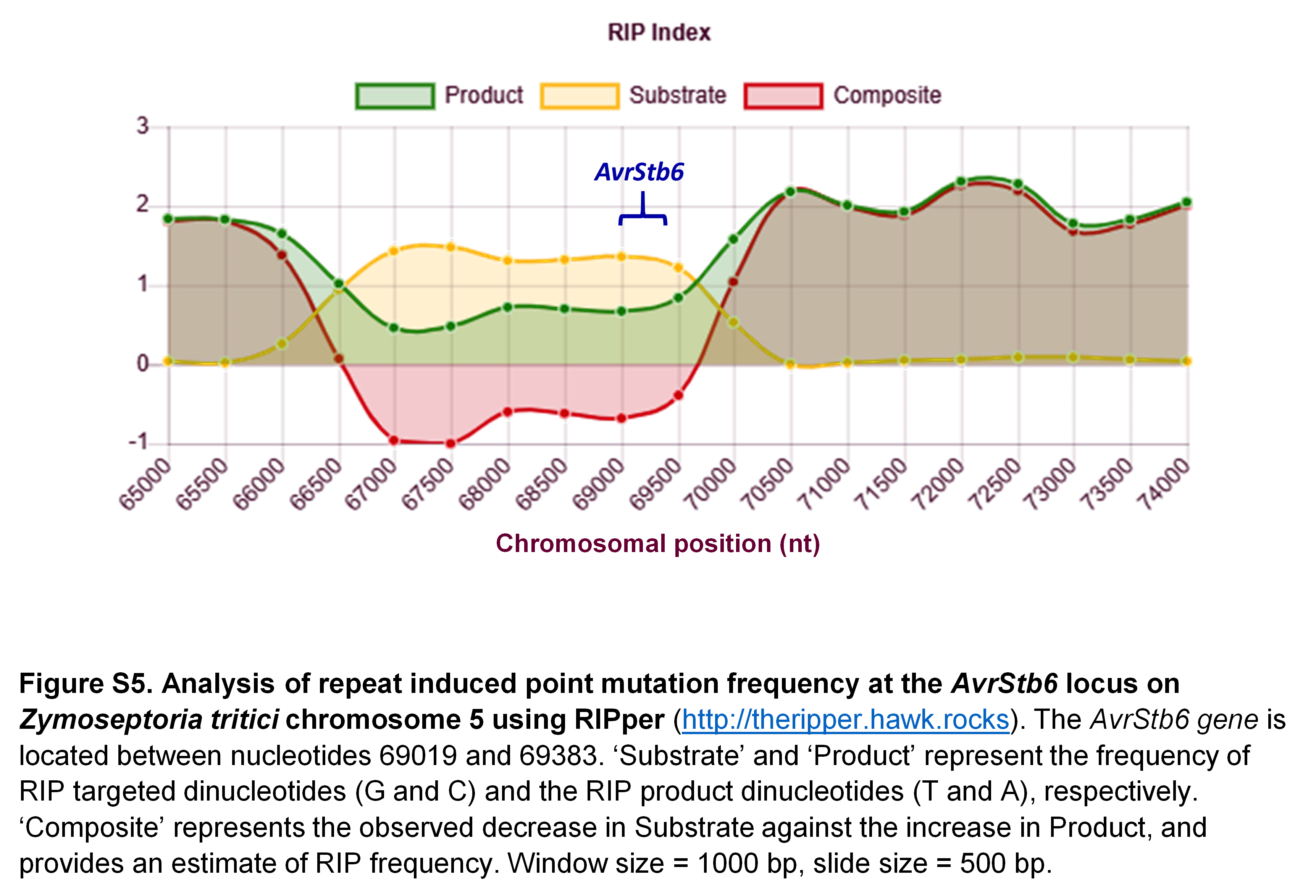

Supplement: Supplementary file 5 — FIGURE S5 Analysis of repeat‐induced point mutation frequency at the AvrStb6 locus on Zymoseptoria tritici chromosome 5 using RIPper (http://theripper.hawk.rocks) [file MPP-22-1121-s001.jpg]
