# Supplementary material for: Remarkable recent changes in the genetic diversity of the avirulence gene AvrStb6 in global populations of the wheat pathogen Zymoseptoria tritici
Source: Mol Plant Pathol. 2021 Jul 14;22(9):1121–33. doi: 10.1111/mpp.13101 (PMC8358995; doi:10.1111/mpp.13101)
Supplement: Supplementary file 4 — FIGURE S4 Expression levels of different AvrStb6 haplotypes during Zymoseptoria tritici infection of wheat [file MPP-22-1121-s007.pdf]

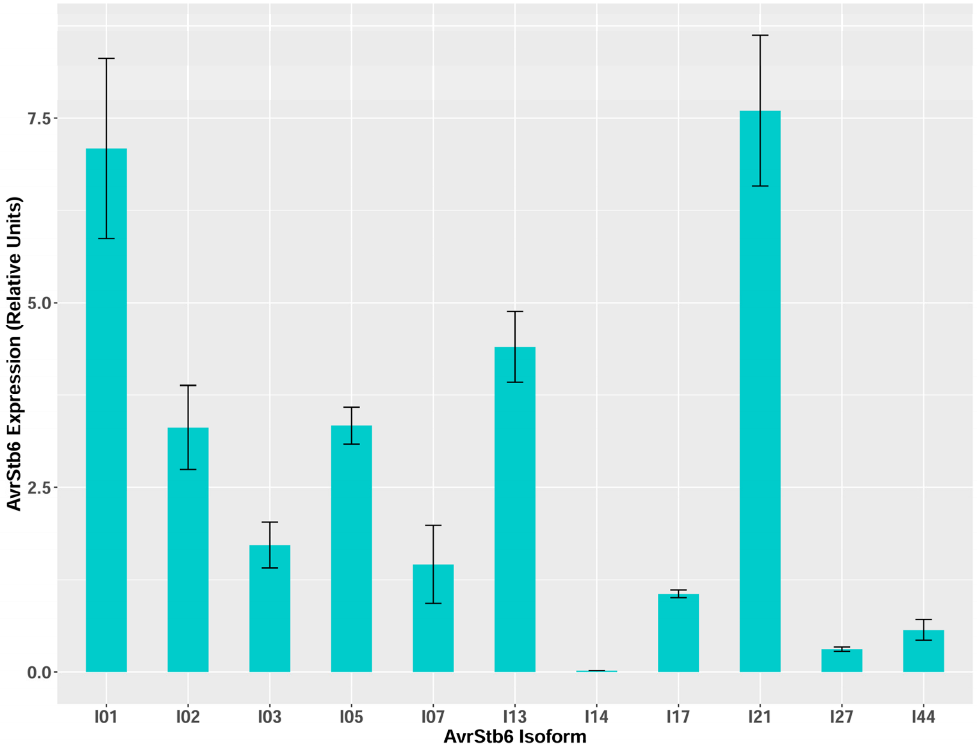


**Figure S4. Expression levels of different *AvrStb6* haplotypes during *Z. tritici* infection of wheat.** Leaves of highly susceptible wheat cv. Taichung 29, containing no known Septoria tritici blotch resistance genes, were inoculated with a selection of *Z. tritici* strains representing different *AvrStb6* haplotypes (giving rise to different protein isoforms) were harvested upon emergence of visible disease symptoms. Error bars are standard errors from three biological replicates. Expression levels are relative to the expression of the housekeeping gene *G6PDH* that encodes glucose-6-phosphate 1-dehydrogenase.
