# Supplementary material for: Remarkable recent changes in the genetic diversity of the avirulence gene AvrStb6 in global populations of the wheat pathogen Zymoseptoria tritici
Source: Mol Plant Pathol. 2021 Jul 14;22(9):1121–33. doi: 10.1111/mpp.13101 (PMC8358995; doi:10.1111/mpp.13101)
Supplement: Supplementary file 6 — FIGURE S6 Alignment of the part of the Stb6 coding sequence (exon 1) with the corresponding region in the CRISPR/Cas9‐induced wheat Cadenza ΔStb6 mutant [file MPP-22-1121-s006.pdf]

```
                                                              target 48f
STB6 WT   1  ATGTCTCTGAGCTGCTGGTCCTGGTTCCTCGCCTTCGCCTGGGTTTGGTGTCTGCCACTG   60
 ΔStb6    1  ATGTCTCTGAGCTGCTGGTCCTGGTTCCTCGCCTTCGCCTGGGTTTGGTGTCTGCCACTG   60

                                                            target 104r
STB6 WT  61  ATGCTCATGGCGGCCGAGGAGCAGCAAGGGGATGGCTGCTTGGCCAAGTGTGGCAGCGTC  120
 ΔStb6   61  A------------------------------------------------GTGGCAGCGTC   72

STB6 WT 121  ACCATCTCCCCCCCGTTCTGGCTCACTGATTGGCAAACAGGAAGATTATGTGGTTCGCCT  180
 ΔStb6   73  ACCATCTCCCCCCCGTTCTGGCTCACTGATTGGCAAACAGGAAGATTATGTGGTTCGCCT  132

STB6 WT 181  GGACCGCTGGACTTCGAGCTTACATGCTATAACGGCAGTTATCCACTTCTTCCAAGCTCT  240
 ΔStb6  133  GGACCGCTGGACTTCGAGCTTACATGCTATAACGGCAGTTATCCACTTCTTCCAAGCTCT  192
                                                        target 278r
STB6 WT 241  GTGCCCAACAACGCCGGCTTTGCAATCATGGACATATCCTATGAGGAACGCAGCTTGCGC  300
 ΔStb6  193  GTGCCCAACAACGCCGGCTTTGCAATCATGGACATATCCTATGAGGAACGCAGCTTGCGC  252

STB6 WT 301  GTCGTTGATCTACGCAAGCTGCAACTATTACACGACCCGCCCAACATCTTCAACAGCTGC  360
 ΔStb6  253  GTCGTTGATCTACGCAAGCTGCAACTATTACACGACCCGCCCAACATCTTCAACAGCTGC  312

STB6 WT 361  TTGCCGATGTGGAACACCTCTGCCAAACTGGGCCGCCCGTTTAAGATCTCCCCCGTCAAC  420
 ΔStb6  313  TTGCCGATGTGGAACACCTCTGCCAAACTGGGCCGCCCGTTTAAGATCTCCCCCGTCAAC  372
                                                          target 465f
STB6 WT 421  CTGGAACTCATCTTGTACAACTGCACGGAGAAGGCCGCCGCGGCGGCACGCCTGGATAAA  480
 ΔStb6  373  CTGGAACTCATCTTGTACAACTGCACGGAGAAGGCCGCCGCGGCGGCACGCCTGGATAAA  432

STB6 WT 481  GAACTGGTGCAGGCGAAGACGATGAGGTGCGTGAACACAAGCAACACGTTTGTTCATGCG  540
 ΔStb6  433  -AACTGGTGCAGGCGAAGACGATGAGGTGCGTGAACACAAGCAACACGTTTGTTCATGCG  491

STB6 WT 541  GGGGTGCCATACGACACCACCGGGACCTACTCTAGTTATGCTTTGGAGGGCTGCGTTCCA  600
 ΔStb6  492  GGGGTGCCATACGACACCACCGGGACCTACTCTAGTTATGCTTTGGAGGGCTGCGTTCCA  551

STB6 WT 601  ATCGTCTTGCCGGTGCTGCGCTTGCCATCCGGCGAGACGAACACGAGCCACTACGAGCGG  660
 ΔStb6  552  ATCGTCTTGCCGGTGCTGCGCTTGCCATCCGGCGAGACGAACACGAGCCACTACGAGCGG  611

STB6 WT 661  CTCATCCAAAGTGGCTTCCTCCTGAAATGGGAACTGCCCCCTCCTCTCCCTGCACCTGCA  720
 ΔStb6  612  CTCATCCAAAGTGGCTTCCTCCTGAAATGGGAACTGCCCCCTCCTCTCCCTGCACCTGCA  671
                 target 715r
STB6 WT 721  CCTAGGAATGAACCACCTCCCCCTCCAG 748
 ΔStb6  672  CCTAGGAATGAACCACCTCCCCCTCCAG 699
```

Figure S6. Alignment of the part of the *Stb6* coding sequence (exon 1) with the corresponding region in the CRISPR/Cas9-induced wheat Cadenza *ΔStb6* mutant. Induced deletions are shaded in red. sgRNA targets are shown in blue. Protospacer adjacent motifs (PAMs) are shown in green. The premature STOP codon in *ΔStb6* is shown in red.
