# Supplementary material for: Remarkable recent changes in the genetic diversity of the avirulence gene AvrStb6 in global populations of the wheat pathogen Zymoseptoria tritici
Source: Mol Plant Pathol. 2021 Jul 14;22(9):1121–33. doi: 10.1111/mpp.13101 (PMC8358995; doi:10.1111/mpp.13101)
Supplement: Supplementary file 8 — TABLE S2 Primers used in this study [file MPP-22-1121-s005.pdf]

Table S2. Primers used in this study.

Primer Name	5' to 3' Sequence	Purpose
avrstb6.f1	CACTTCTTTCCACAACTCCCACTT	Amplification and sequencing of *AvrStb6*
avrstb6.f3	ATCAACTTCCTCTCAACCAAGACC	
avrstb6.r1	CCTACATTGGCAGCATCAAAATCA	
8311F19	CGCGGTTCCAGTCACATCAC	Amplification and sequencing of *Stb6*
8311F3	CCGTTTAGCTCGTGTTGTGC	
8311R5F	CTGGACCGCTGGACTTCGAG	
1186R2	GAGCAAGCTTTCAATTACAGGAG	
13609F1	CTGAAAAAAAATACGAGGCCATGA	
8311F16	GCGACATGGTAGCTCAATCAAA	
8311R16	TTCCTTCCATGGTCGGTAACTT	
JPG G6PDH F1	GCGGCTACTTTGACGAGTTC	RT-qPCR analysis of *AvrStb6* expression
JPG G6PDH R1	GATCCGTCAAGCGACTTCTC	
AvrStb6 F6b	TTCTACAAGGCTTCCTCGC	
AvrStb6 R6b	GCTTTCCGTCGTGGCAGAA	
